# From Forces to Assemblies: van der Waals Forces-Driven Assemblies in Anisotropic Quasi-2D Graphene and Quasi-1D Nanocellulose Heterointerfaces towards Quasi-3D Nanoarchitecture

**DOI:** 10.3390/nano13172399

**Published:** 2023-08-23

**Authors:** Abdelrahman Brakat, Hongwei Zhu

**Affiliations:** State Key Laboratory of New Ceramics and Fine Processing, School of Materials Science and Engineering, Tsinghua University, Beijing 100084, China

**Keywords:** van der Waals (vdW) forces, heterointerfaces, anisotropic, graphene, nanocellulose, nanoarchitecture

## Abstract

In the pursuit of advanced functional materials, the role of low-dimensional van der Waals (vdW) heterointerfaces has recently ignited noteworthy scientific interest, particularly in assemblies that incorporate quasi-2D graphene and quasi-1D nanocellulose derivatives. The growing interest predominantly stems from the potential to fabricate distinct genres of quasi-2D/1D nanoarchitecture governed by vdW forces. Despite the possibilities, the inherent properties of these nanoscale entities are limited by in-plane covalent bonding and the existence of dangling π-bonds, constraints that inhibit emergent behavior at heterointerfaces. An innovative response to these limitations proposes a mechanism that binds multilayered quasi-2D nanosheets with quasi-1D nanochains, capitalizing on out-of-plane non-covalent interactions. The approach facilitates the generation of dangling bond-free iso-surfaces and promotes the functionalization of multilayered materials with exceptional properties. However, a gap still persists in understanding transition and alignment mechanisms in disordered multilayered structures, despite the extensive exploration of monolayer and asymmetric bilayer arrangements. In this perspective, we comprehensively review the sophisticated aspects of multidimensional vdW heterointerfaces composed of quasi-2D/1D graphene and nanocellulose derivatives. Further, we discuss the profound impacts of anisotropy nature and geometric configurations, including in-plane and out-of-plane dynamics on multiscale vdW heterointerfaces. Ultimately, we shed light on the emerging prospects and challenges linked to constructing advanced functional materials in the burgeoning domain of quasi-3D nanoarchitecture.

## 1. Introduction

The phenomenal evolution of nanotechnology has paved the way for significant progress in advanced functional materials, seamlessly bridging the gap between nanoscopic and microscopic domains with its macroscopic realm. An integral part of this progression has been the ingenious employment of top–down synthesis and bottom–up assembly strategies, enabling the creation of high-quality, low-dimensional building blocks with unique properties. Among these, graphene 2D nanosheet [1,2,3,4] and nanocellulose (NC) 1D chain [5,6,7,8] derivatives are gaining significant attention, primarily driven by their exceptional physicochemical properties [5,9,10,11,12] and the prospects they hold for metamorphosing into multidimensional bio-inspired hierarchical structures [6,13,14,15,16,17,18,19,20,21,22,23,24,25,26]. The tantalizing range of potential applications, spanning from multi-sensing [27,28,29,30,31,32,33,34,35,36] and energy storage [37,38] to insulation [24,39], shielding [40,41], and environmental remediation [23,42,43,44,45], accentuates a significant role across multiple disciplines.

Despite the remarkable paces in nanotechnology, numerous challenges overshadow the path towards fabrication of isolated, pristine, monodispersed 2D nanosheet and 1D nanochain. This is particularly true given the inter–intramolecular structures [9,46] and hydrophilicity–hydrophobicity (OH-π/CH-π domains and (110)/(010)/(1$\bar{1}$0) faces) [2,47,48,49,50,51] these aromatic quasi-2D and aliphatic quasi-1D nano-entities possess. Herein, the terms quasi-2D and quasi-1D indicate that these structures are nearly, but not perfectly, two-dimensional and one-dimensional, likely having imperfections as defects or branching that deviate from a perfectly flat plane hexagonal lattice or tetrahedral geometry, respectively. Among the multitude of challenges, the reactive center–basal–edge plane of quasi-2D monolayer, with dangling π-bonds [50,52], can initiate undesired covalent bonds, thereby compromising the structural integrity [52]. Moreover, quasi-2D bi–tri–multilayers tend to form π-π stacking interactions [53,54], impeding the pristine electronic structures and uniform dispersion forces in solvents or matrices [55]. Similarly, quasi-1D NC chains, with abundant oxygen functional groups (i.e., –OH groups), are susceptible to strong covalent interactions, leading to agglomeration [5,56]. This can disrupt the assembly process and impede the functionality of the nanochain [56]. It is also worth noting that defects in the graphene quasi-2D monolayer, as imperfections or the ratio of ordered to disordered within the sp^2^ and sp^3^ hybridized domains [57], could interfere with its desirable electronic and thermal attributes [57,58,59].

In spite of the multitude of challenges, the interplay of weak inter–intramolecular forces in close proximity [60,61], namely, van der Waals (vdW) forces [62,63,64], serves as the driving force behind dynamic non-covalent interactions [65,66]. This presents an intriguing opportunity to manipulate these quasi-2D/1D building blocks at heterointerfaces [8,50,63,67]. Although seemingly weak in nature, these forces are integral to the field of nanoarchitectonics [68,69,70]. They play a significant role in assembling quasi-2D/1D building blocks across heterogeneous interfaces [71,72,73], leading to the evolution of quasi-3D nanoarchitecture. Capitalizing on this understanding, scholars have leveraged the principles of interfacial nanoarchitectonics to drive the self-assembly of nanoscale quasi-2D/1D building blocks into an organized hierarchical architecture with desirable attributes [73,74]. By manipulating assembly conditions, kinetics, and driving forces, including the presence of specific solvents, such as ionic liquids (ILs) [75,76,77], we can influence the strength and orientation of these vdW forces. This affords precise control over the assembly process through vdW-driven assembly approaches, precisely layer-by-layer assembly [78], and freeze-casting drying [19]. Moreover, the amphiphilic nature of quasi-2D/1D interfaces [79,80], derived from OH-π and CH-π domains, presents an opportunity for generating hydrogen bonds [51,66,79]. Consequently, introducing an anisotropic quasi-1D NC chain [81,82,83,84] onto quasi-2D graphene nanosheets [85] offers further opportunities through vdW-driven functionalization. By employing covalent and non-covalent functionalization strategies [65,67,86,87], these nanostructures can be finely tuned to exhibit unique properties. Also, the creation of reinforcing linkages through chemical or physical crosslinking sites [85] not only strengthens the inter–intrachain and inter–intrasheet intrinsically [49,51], but also extends practical applicability [24,43,88,89,90]. Therefore, it is particularly noteworthy that these vdW forces uniquely define load transfer (LT), charge transfer (CT), and heat transfer (HT) mechanisms at the quasi-2D/1D heterointerfaces, whether combined or separated. Progress has been made experimentally [5,47,49,51,57,82,91,92,93] and computationally [51,79,92,94,95,96,97,98,99,100] in the single-scale regime, specifically, in the context of quasi-2D graphene mono–bi–trilayer and quasi-1D NC isolated single–multichain. However, significant gaps still persist in our understanding of the vdW-driven transition in multiscale regimes, such as quasi-2D/1D multilayer. Additionally, the in-plane and out-of-plane dynamics within these nanoarchitectures need comprehensive comprehension. For instance, significant empirical and computational findings in the vdW-driven transition underscore them in terms of in-plane properties [24,39,53,91,94,101]. In some cases, a balance between bonded and non-bonded forces dictates the LT mechanism, mirroring properties of the high elastic modulus matching Kevlar [102], attributed to anisotropic quasi-1D nanocrystal features and structured hydrogen bonds [94,101]. However, out-of-plane properties [101] are mainly directed by vdW forces, significantly impacting the strength and elasticity of multiscale structures. Despite progress made, there is still a need for further exploration of deformation behavior under various load conditions, interactions between vdW forces and atomic-level forces, and the role in anisotropic quasi-1D NC rod-like chain micromechanics of vdW heterointerfaces [49]. While we understand the role of quasi-2D sp^2^ and quasi-1D NC sp^3^ hybridized domains in the CT mechanism (i.e., hopping [103,104] and overlapping [91]), further research is needed to fully grasp in-plane and out-of-plane electrical conductivities within these multiscale nanoarchitectures. Even though the superior in-plane thermal conductivity (K_||_ λ_X_) of anisotropic quasi-2D nanosheet is well understood [39,105,106,107], there is a need for further research regarding the out-of-plane thermal conductivities (K_⊥_ λ_Z_) in the context of anisotropic quasi-1D NC chain [108,109,110,111]. Specifically, the potential for thermal management applications has yet to be fully explored [109,110,111,112]. Consequently, gaining a comprehensive understanding of the vdW-driven transition is crucial for optimizing the efficiency and unlocking the full potential of these multiscale nanoarchitectures [19,24]. Recent findings emphasize the crucial role of vdW forces in aligning quasi-2D/1D heterointerfaces into a quasi-3D nanoarchitecture. For instance, when monodispersed colloids are subjected to in situ protocols, namely, over-compression, they can trigger phase transitions, modify isothermal surface pressure, and aid in constructing 3D multilayer structures [113,114,115,116]. In addition, ex situ protocols, such as electromagnetic [117], acoustic [118], or light-irradiation [119] external fields, can manipulate interfacial interactions and configurations, leading to highly ordered quasi-3D porous structures [19,120]. These advances facilitate the conversion of smooth quasi-2D/1D surfaces into 3D porous assemblies, unveiling advanced functionalities, including nanoporosity for thermal insulation [24] or separation [44] technologies. However, further exploration is particularly required in the context of vdW-driven alignment. Intriguingly, by leveraging the anisotropic helical features inherent in quasi-2D/1D heterointerfaces, monodispersed liquid crystals (LCs) can be transformed into helical orders similar to chiral nematic [5,121,122,123,124,125]. This transformation is primarily driven by vdW forces, which promote alignment and reduce free energy. The resulting tailored properties open up opportunities in fields such as optics [126], photonics [121,127], and sensing [125] technologies.

In this perspective article, our objective is to provide a comprehensive roadmap that elucidates the potential of vdW-driven interfacial interactions in assembling and transforming quasi-2D graphene and quasi-1D nanocellulose derivatives into quasi-3D nanoarchitectures. Initially, we will explain the fundamental aspects of vdW forces and functionalization, demonstrating the transformative impact they have on load, charge, and heat transfer mechanisms at heterointerfaces. Following this, we will elaborate on the metamorphosis of these vdW heterointerfaces into quasi-3D nanoarchitectures, a process facilitated by vdW-driven alignment. Ultimately, through the prospective roadmap, we aim to inspire further research in this dynamic domain and shed light on its wide-ranging implications across multiple disciplines.

## 2. vdW-Driven Assembly

**Figure 1:**
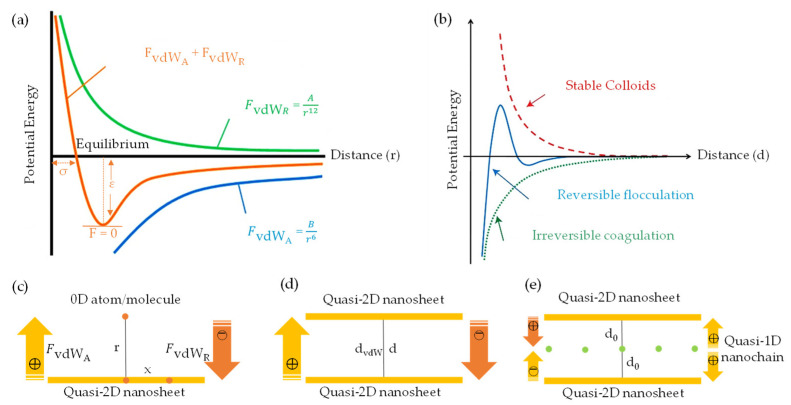
(**a**) Lennard−Jones potential ($U_{LJ}$) curve versus separation distance (*r*) [130]; (**b**) Potential energy (U) versus separation distance (*d*) profiles for classical types of DLVO colloidal stability theory [131]. Reproduced with permission. Copyright: American Chemical Society. (**c**–**e**) Schematic representation of potential vdW attractive and repulsive forces interactions between low-dimensional building blocks based on the separation distance (*r*/*d*/*x*) and interlayer spacing (d, d_0_, d_vdW_).

The vdW-driven assembly strategy has been effectively employed to explore the interaction among 0D, 1D, and 2D building blocks. This strategy harbors significant potential for fabricating innovative materials and devices possessing unique properties. In this context, low-dimensional building blocks derived from graphene and nanocellulose demonstrate intriguing characteristics, propelling widespread use in a diverse range of applications, from nonelectrochemical to nanoelectronics systems. With the advent of advances in top–down synthesis routes, highly sophisticated quasi-2D/1D building blocks could become realizable. This possibility has spurred further exploratory studies to assemble macroscopic nanoarchitecture. Fundamentally, the electrostatic stability of monodispersed colloids is dictated by the sum of vdW attractive ($F_{\mathrm{vdW}A}$) and repulsive ($F_{\mathrm{vdW}R}$) forces, in addition to the separation distance (*r*/*d*) at equilibrium between atoms, molecules, or nano-entities (Figure 1a,b). This concept is based on the Derjaguin–Landau–Verwey–Overbeek (DLVO) theory ($F_{{\mathrm{vdW}}_{\mathrm{DLVO}}}$) [128] and the Lennard-Jones potential (*U_LJ_*) [129,130]. It is important to note that vdW attraction and repulsion are inversely proportional to the 6th/12th power of (*r*/*d*).

(1) $$\;{F_{{\mathrm{vdW}}_{\mathrm{DLVO}}}=F}_{{\mathrm{vdW}}_{A}}+F_{{\mathrm{vdW}}_{R}}$$

(2) $${\;\;\;\;\;\;\;\;\;\;\;\;U}_{LJ}=\frac{A}{r^{6}}-\frac{B}{r^{12}}$$

**Figure 2:**
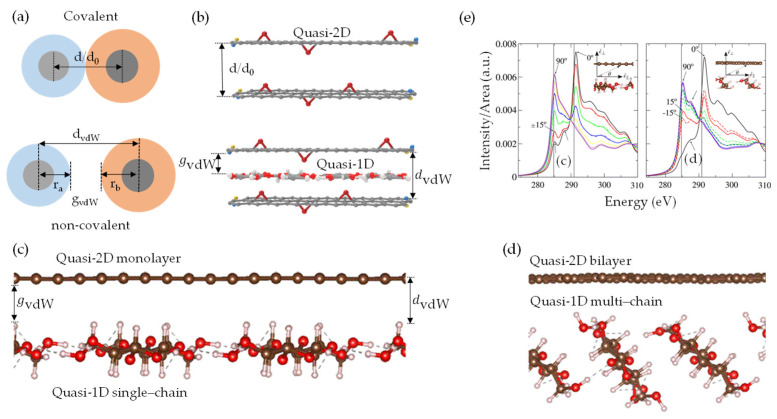
(**a**,**b**) Schematic representation of vdW gap (g_vdW_) in covalent and non-covalent bonds based on the molecules or objects radii (r_a_/r_b_), vdW distance (d_vdW_), and interlayer spacing (*d*/d_0_) [138]. Modified with permission. Copyright: Nature. (**c**–**e**) Binding energy relative to the equilibrium geometry shown for the respective quasi-2D/1D structural models of mono–bilayer and single–multichain, with iso-surfaces and interface regions depicted [51]. Reproduced with permission. Copyright: Royal Society of Chemistry.

When multiple atoms, molecules, or nano-entities (i, j) approach each other at a certain distance (*r*, *x*, *d*, d_0_, or d_vdW_), as depicted in Figure 1c, inter–intramolecular forces denoted as $F_{{\mathrm{vdW}}_{A}}$ and $F_{{\mathrm{vdW}}_{R}},\;$respectively, interact due to electronic polarizability [60]. This interaction generates potential energy (U), which is governed by U_LJ_ parameters (A, B) and specific geometric parameters. Within the scope of quasi-2D/2D vdW heterointerfaces, the interfacial interaction between two quasi-2D heterointerfaces (Figure 1d) can be comprehended by considering the fundamental geometric configurations. These configurations encompass face-to-face or face-to-edge encounters occurring at the basal–center–edge planes. The shape of these interfaces ranges from nanometers to micrometers on the lateral scale, while the thickness is defined by a single atom layer, rendering them an unconventional soft nanomaterial [132]. Based on the DLVO theory (Figure 1b), a range of scenarios, including stability, flocculation, and coagulation, may arise upon the interaction between dissimilar quasi-2D/2D monodispersed colloids with large aspect ratios, anisotropic shapes, and varying surface areas [128,131]. To be specific, coagulation and flocculation occur in all monodispersed colloids due to the presence of vdW attraction and repulsion, respectively. When a colloidal suspension is kinetically stabilized solely by electrostatic attraction or repulsion, it can undergo either weak irreversible coagulation or reversible flocculation. This phenomenon leads to the formation of double electrical layers, which introduce attractive and repulsive energy curves. Consequently, the quasi-2D/2D colloid would not be stable and tends to stack together with a vdW potential scale of $\left(\frac{1}{d^{n}}\right)\;$ [128]. Electrostatic stabilization of colloids, involving biopolymer chain-like quasi-1D NC or ionic liquid-derived surfactants [133,134], represents a potential strategy for limiting flocculation and coagulation phenomena. These substances can effectively impart a high surface density charge or zeta potential and specific interlayer distance (d_0_ or d_vdW_), contributing to the consistent electrostatic stability of colloids (stably charged) [128]. For example, colloidal suspensions with a zeta potential value greater than 30 mV or less than −30 mV, depending on the nature of the surface charge, are generally considered solely stable [135]. Correspondingly, the efficacy of the organic ILs in creating a polar environment has been found to be favorable for the dispersion and stabilization of quasi-2D/1D colloids compared to other dispersants and stabilizers [136,137]. Consequently, the stability of such a colloidal suspension is determined not only by the interplay of vdW attraction and electrostatic repulsion forces, but also by the geometric relationship between the interacting nano-entities [60]. Principally, traditional U_LJ_ is frequently used to simulate vdW interactions. However, the non-linear nature can lead to inaccuracies when applied to complex systems, such as quasi-2D/1D heterointerfaces. Concerning vdW interactions [61,62], parameters such as the vdW distance (d_vdW_) and vdW gap (g_vdW_) [138] are essential for determining the binding energy and forces associated with bonded or non-bonded interactions between atoms, molecules, or nano-entities (Figure 2a), as prescribed by Equation (3).

g_vdW_ ≈ d_vdW_ − r_a_ − r_b_ (3)

where r_a_, r_b_ are individual atoms, molecules, or nano-entities radii (g_vdW_ < *d*/d_0_). The vdW gap refers to the separation distance between two interacting nano-entities, where the vdW interaction energy reaches a minimum [62]. This minimum energy corresponds to the most stable configuration of the system and is referred to as the equilibrium distance or the vdW distance (d_vdW_). Consequently, the crucial role of long-range or short-range vdW interactions in the binding energy between quasi-2D/1D heterointerfaces is significant (Figure 2b), intersheet and interchain [49,51]. Within this framework, the d_vdW_ and g_vdW_ are typically defined using a pairwise potential function, such as the U_LJ_ [130], which describes the interaction energy between two nano-entities as a function of the separation distance (*r*). For example, in U_LJ_, the interaction energy between two non-bonding nano-entities is represented by a specific formula:

(4) $$U_{LJ}=4\varepsilon \left[{\left(\frac{\sigma }{r}\right)}^{12}-{\left(\frac{\sigma }{r}\right)}^{6}\right]$$

where *r* is the distance between two entities, ε is the depth of the potential well, and σ is the distance at which the potential energy equals zero. The g_vdW_, in this case, is defined as the value of σ. In the assembly driven by vdW forces, the g_vdW_ is typically chosen to align with theoretical or experimental values for the d_vdW_. It also influences the convergence and efficiency of the numerical integration method to compute the vdW energy. In a comprehensive computational study by Silvestre et al. [51], the authors utilized DFT simulations considering the vdW approach [139] to elucidate the significance of vdW interactions in governing the bonded or non-bonded interactions at hydrophilic–hydrophobic quasi-2D/1D heterointerfaces (Figure 2c,d). The primary findings of the study underscore the pivotal role of vdW-driven assembly in the interfacial interactions between quasi-2D graphene nanosheet and quasi-1D NC chain. This is evident in the binding energy and vertical distance (h:d_vdW_) calculations for the quasi-2D/1D interface. Remarkably, the study offers a quantitative analysis of the binding energy (E_binding_) by comparing the total energy of the integrated system E_quasi-2D/1D_ with the combined energies of the individual components E_quasi-1D_, E_quasi-2D_, following the formula:

E_binding_ = E_quasi−2D/1D_ − E_quasi−1D_ − E_quasi−2D_ (5)

where E_quasi-1D_ is a singular sheet of NC nanofibrils, and E_quasi−2D_ is G monolayer binding energies. The binding energy, calculated through the vdW-DF approach [140] (Figure 2e), amounted to −12.92 and −11.63 meV Å^−2^ for hydrophilic–hydrophobic interfaces, respectively, demonstrating an energetic preference for the hydrophobic interface. Furthermore, the vertical distance (h) between the graphene mono–bilayer and NC single–multichain exhibited minimal changes, less than 0.01 Å. Also, by employing an implicit solvation model, a decrease in interface binding energy was noted, which was more pronounced for the hydrophilic interface due to its nature, corroborating solvation energy results. This importance is further supported when neglecting the vdW contribution; the E_binding_ (−0.51) (−0.81) meV Å^−2^ and h 3.04 (2.90) Å dropped drastically, suggesting that non-covalent vdW interactions primarily govern the formation of quasi-2D/1D interfaces. Therefore, the crucial role of vdW forces in the dynamic interaction between quasi-2D nanosheet and quasi-1D nanochain is unequivocally established.

**Figure 3:**
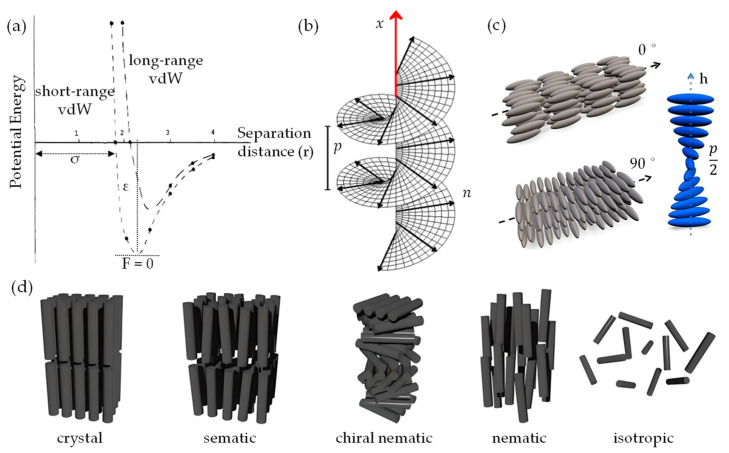
(**a**) Lennard-Jones potential curve of the short- and long-range chiral vdW interactions (like/unlike) with separation distance (*r*) [142]; (**b**) Schematic view of liquid crystals (LCs) with a distinct helical feature along chiral nematic directors (*x*, *n*) and pitch (*p*) [144]. Reproduced with permission. Copyright: American Physical Society. (**c**) Parallel, perpendicular, and chiral nematic configurations of half-pitch ($\frac{p}{2}$) quasi-1D NC rod-like crystals concerning the shear and helical directions [145]. Reproduced with permission. Copyright: American Chemical Society. (**d**) Schematic view illustrating the LCs phase transition and self-assembly into sematic–chiral nematic–nematic orders upon lyophilization governed by certain concentration and temperature [121]. Reproduced with permission. Copyright: WILEY-VCH.

Notably, when considering the features of anisotropic monodispersed liquid crystals (LCs), it is crucial to note the myriad forces acting upon them, including vdW forces and steric repulsion. The vdW forces, which are attractive in nature, promote the congregation of nano-entities. Conversely, steric repulsion serves as a counterforce, preventing these nano-entities from approaching too closely [64]. Consequently, an orientation alignment of nano-entities emerges, effectively reducing the free energy. These interactions stimulate the formation of self-assembled and highly ordered structures within the LCs. As the constituent nano-entities initiate this process of structural self-organization, a concomitant decrease in free energy driving this assembly progresses even further [141]. Nevertheless, several researchers have explored the application of modified U_LJ_ to simulate vdW interactions among chiral nano-entities [142]. A particular illustration is the chiral U_LJ_ that integrates an extra term to compensate for the chiral interactivity between the nano-entities, as depicted in Figure 3a. The potential energy curve for the chiral U_LJ_ can be written as follows:

(6) $$U_{LJ}=4\varepsilon \left[{\left(\frac{\sigma }{r}\right)}^{12}-{\left(\frac{\sigma }{r}\right)}^{6}\right]+\Delta U_{LJ}\;$$

where the extra term ΔU_LJ_ accounts for the chiral interactions between the nano-entities; the shape of ΔU_LJ_ depends on the specific form of the chiral interactions, but it typically introduces a directional dependence to the potential energy. As illustrated in Figure 3b, chiral nematic LCs exhibit a distinctive property whereby the nematic director (*n*) undergoes helical rotation around a chiral director (*x*), resulting in a chiral distribution of nano-entities orientations [143,144,145].

At certain concentrations (wt.%) or volume fractions (φ) and equilibriums of monodispersed LCs [121], there is a probability of self-assembly and a transition from liquid to solid state. This could lead to the formation of specific sematic, nematic, and chiral nematic ordered structures [121,125,143,145] upon lyophilization (Figure 3c,d). Experimentally, d_vdW_/g_vdW_ between quasi-1D NC chains can vary, depending on the specific conditions and the degree of polymerization of the nanochain. For instance, atomic force microscopy (AFM) studies [63,146] have reported a d_vdW_/g_vdW_ of less than 10 nm between individual NC chain binding domains, which exhibit a rod-like shape, with diameters ranging from 3 to 10 nm and lengths of several hundred nanometers. Alternatively, the d_vdW_/g_vdW_ in the case of quasi-2D nanosheets arranged in a stacked configuration refers to the interspace where the attractive vdW forces counterbalance the electrostatic repulsion between the sheets. The size of the d_vdW_/g_vdW_ can vary, depending on the number of layers and the degree of oxidation of the graphene nanosheet. For instance, experimental findings indicate a d_vdW_/g_vdW_ ranging from 0.39 to 0.42 nm for graphene oxide (GO) nanosheet composed of 3–4 layers [147]. Yet, for a thinner sheet comprised of 1–2 layers, the d_vdW_/g_vdW_ may diminish to approximately 0.34 nm [147]. Thus, the fundamental concepts within the vdW-driven assembly in quasi-2D/1D heterointerfaces depend highly on multiple parameters, including morphology, size, surface chemistry, and environmental factors. It is evident that tailoring surface functionalities can substantially alter these vdW forces, as these changes can impact the electronic and chemical attributes and, in turn, modify the intensity of these vdW interactions. Precise assessments of these aspects are imperative for molecular dynamic (MD) simulations to adequately depict the behavior and characteristics of nano-entities when they are in close proximity to each other. Despite the progress made, further in-depth exploration is crucial to fully grasp the repercussions of these vdW interactions.

## 3. vdW-Driven Functionalization

Gaining insights into interfacial interactions within the heterointerface is crucial for effectively structuring and tailoring the functionalities of nanoarchitecture [138]. Graphene and nanocellulose derivatives, in quasi-2D inter–intrasheet [148] or quasi-1D inter–intrachain [83,149] configurations, possess undesirable characteristics owing to the atomic and molecular structures, namely, the concept of dangling bonds or dangling π-bonds [50,52]. These are bonds where an atom (like carbon in graphene) has an unpaired electron in its outermost shell that can form a π-bond available for bonding with other atoms (like oxygen or hydrogen).

**Figure 4:**
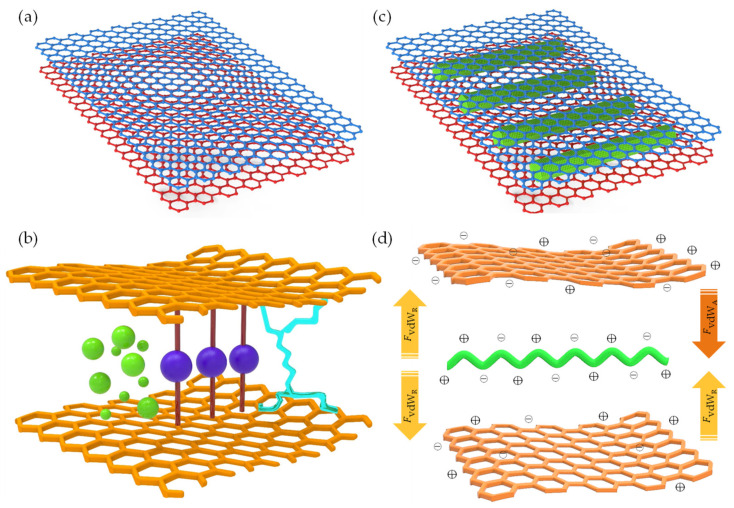
Schematic representation of low-dimensional building blocks binding with vdW forces interactions (surface functional groups have been omitted for clarity): (**a**,**b**) quasi-2D/2D graphene heterointerface with possible overlayer binding and coupling capabilities driven by dangling π-bonds (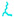), hydrogen bonds (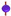), and π-conjugated molecules (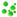); (**c**,**d**) quasi-2D/1D vdW heterointerfaces and proposed weak-synergy interactions driven by vdW forces.

This dangling π-bond is delocalized across the entire nanosheet, creating the characteristic π-conjugation that gives graphene many unique properties, such as its high electrical–thermal conductivities. However, these π-bonds can also “dangle” at the edges, creating reactivity similar to dangling bonds (Figure 4a,b). These highly reactive bonds offer both opportunities and challenges, being able to chemically adjust the surface and bond to other structures, but also leading to potential instability or unwanted reactions under certain conditions. Therefore, the concept of dangling bond-free nanoarchitecture [150,151], along with weak-synergy vdW interactions, holds immense promise in achieving enhanced functionalities of quasi-2D/1D heterointerfaces. A key benefit of these structures is the stability they impart, circumventing the potential for unwanted reactions or instability typically associated with the reactive dangling bonds. For example, the hydrogen passivation of quasi-2D graphene enables the formation of a stable, hydrogen-terminated edge, effectively eliminating the reactive dangling π-bonds [152]. Similarly, quasi-2D/1D surfaces can be chemically modified via ILs [77,86,153]. This modification can be attributed to the anion/cation species in ILs that act as donor–acceptor pairs, disrupting the robust hydrogen-bonding networks through electrostatic interactions [75,134], rendering them dangling bond-free and potentially augmenting the interaction capabilities. Hence, incorporating quasi-1D NC chains and ILs could prove pivotal in amplifying the properties of dangling bond-free quasi-2D graphene (Figure 4c,d). This enhancement might be achievable through dispersion, consequently preventing π-π driven stacking and fortifying the inherent strength and flexibility of NC, thereby boosting its mechanical properties [86,154,155,156].

**Figure 5:**
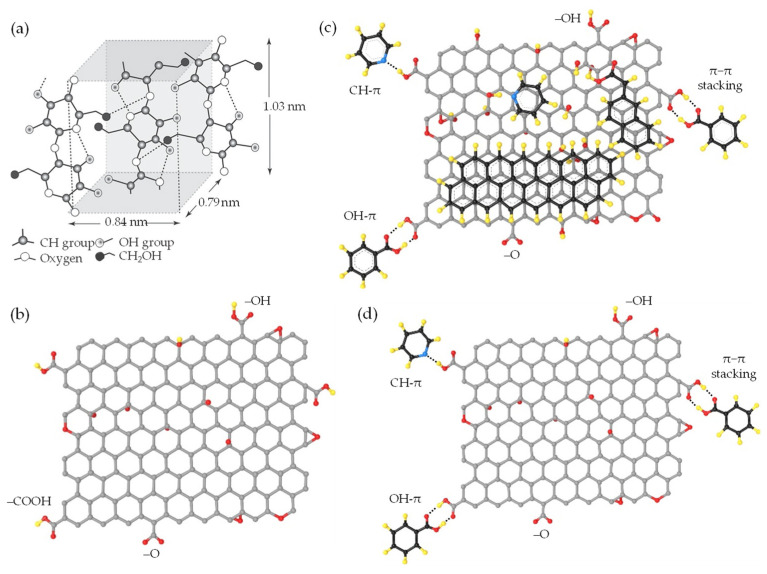
(**a**) Schematic model of quasi-1D NC biopolymer interchain (solid line) and intrachain (dash line) hydrogen bonds [46]. Reproduced with permission. Copyright: The Royal Society. (**b**–**d**) Schematic models of quasi-2D graphene derivatives depicting intersheet covalent interactions (solid line) and intrasheet non-covalent interactions (dash line) [67]. (red: O, white/yellow: H, black/grey/blue: C). Modified with permission. Copyright: American Chemical Society.

As aforementioned in Section 2, the unique properties of quasi-2D/1D heterointerfaces are derived from the interplay between vdW forces ($F_{\mathrm{vdWA}}$/$F_{\mathrm{vdWR}}$) and electrostatic stabilization, as well as the synergistic, weak, non-covalent interactions that are either naturally occurring or bio-synthetically engineered. These forces can play a role in the self-assembly and adhesion of nanosheet and nanochain during the preparation of anisotropic interfacial nanoarchitecture. The phenomenon of non-covalent functionalization, particularly in the context of quasi-2D/1D vdW heterointerfaces, opens up new potential applications. Primarily, the strong synergy between the extensive surface areas of isolated quasi-1D NC chain (Figure 5a) [46] and quasi-2D graphene derivatives nanosheet (Figure 5b) [65,67] is attributed to in-plane covalent bonding interactions. Specifically, these interactions involve inter–intramolecular hydrogen bonds from the plentiful oxygen-containing functional groups/moieties, such as –O, –OH, and –COOH. In contrast, the out-of-plane weak synergy is associated with non-covalent interactions, including vdW forces, hydrophilic–hydrophobic π-domains, and π-π stacking interactions [67]. Furthermore, in-plane and out-of-plane π-π stacking interactions at the basal–edge planes may occur due to weak non-covalent interactions (Figure 5c). This complex interplay primarily stems from the donor–acceptor mechanism facilitated by surface functional groups, which significantly influence the self-assembly of quasi-2D nanosheets. Notably, a marked propensity exists for defects along the basal aromatic quasi-2D nanosheet [100], attributable to carbon atoms bonding with functional groups, such as epoxides or hydroxides. These bonds significantly alter the electronic, electrical, and optical properties of the nanosheet [100]. It is important to underscore the ambiguity in the qualitative and quantitative distribution of oxygen-containing functional groups and π-π stacking within the in-plane and out-of-plane quasi-2D nanosheet. This indicates a necessity to diminish the degree of structural disorder (Figure 5d). Intrinsically, manipulating in-plane and out-of-plane vdW non-covalent interactions within the context of quasi-2D/1D multilayer appears to be a vital aspect when it comes to controlling structural defects and interlayer spacing [65,67].

**Figure 6:**
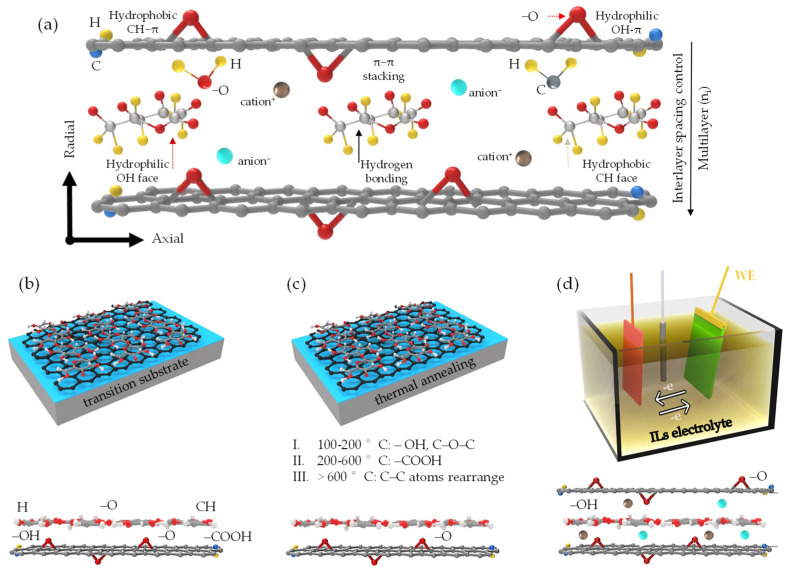
(**a**) Proposed model of the in-plane covalent and out-of-plane non-covalent interactions between quasi-2D/1D inter–intrasheet and inter–intrachain in axial and radial directions (red: O, white/yellow: H, black/grey/blue: C). Schematic representation of in situ reduction mechanisms at quasi-2D/1D heterointerfaces, including (**b**) quasi-1D NC chain; (**c**) thermal annealing; and (**d**) electrochemical reduction.

Covalent functionalization [5,65,157] encompasses the creation of stable and specific chemical bonds within the quasi-2D/1D functional groups, although this procedure often modifies the intrinsic properties of the materials, yielding tunable electronic structures and chemical reactivity. However, it may interfere with the domains that are sp^2^ hybridized, potentially degrading their exceptional electronic properties. In situ chemical [158,159,160], thermal annealing [160,161,162], and electrochemical [162,163] reduction mechanisms have been experimentally verified to eliminate unnecessary in-plane and out-of-plane functional groups and to partially restore the sp^2^ carbon network of the pristine graphene, albeit with some minor defects [158,164]. However, numerous approaches typically require hazardous reductants [165,166] and specific conditions [161,167]. Therefore, preserving the pristine properties of quasi-2D/1D heterointerface with minimal structural damage through non-covalent functionalization could be a feasible approach, yielding excellent multiscale charge and thermal transport pathways [91,93]. Delving deeper into vdW-driven functionalization, the interactions among quasi-1D NC inter–intrachain hydrogen bonds featuring dense hydrophilic OH and hydrophobic CH faces can effectively mediate molecular rearrangement and alignment at quasi-2D/1D heterointerfaces. This is possible in the presence of green organic ILs, as depicted in the proposed model (Figure 6a). The synergistic effect of quasi-1D filler (Figure 6b) has been verified to preclude stacking and enrich the interlayer spacing between adjacent quasi-2D nanosheets [51,92], primarily facilitated by the ordered crystalline structure and anisotropic nature [49]. In a quasi-1D/2D multilayer structure, the heterointerface exhibits a competitive affinity with bonded and non-bonded interactions [90,94] until equilibrium is reached. These inter–intrasheet and inter–intrachain configurations function as efficient conductive pathways, promoting reduction processes through enhanced π-electron transfer in axial and radial directions [51]. This, in turn, facilitates precise control over the π-electron delocalization and dimensionality (spacer) of reduction reactions. Furthermore, thermal annealing processes within a temperature range of 100–600 °C (Figure 6c) can activate in situ reduction at the quasi-2D/1D interface [160]. These reactions effectively evaporate solvents or reagents and remove less thermally stable groups (C–O–C), thereby partially reestablishing sp^2^ hybridized –C– domains. Also, the act of leveraging the distinctive characteristics of quasi-2D graphene flat nanosheet [168] and quasi-1D NC hollow structure [83,169] within ILs electrolyte [170,171] holds substantial value (Figure 6d). Potential reactions between the quasi-2D/1D working electrode and ILs electrolyte may involve groups like –O, –OH, –COOH from the quasi-2D/1D being added to ILs^+^; meanwhile, ILs^−^ loses oxygen atoms (Equations (7) and (8)). The proposed in situ electrochemical reaction mechanism may enable the elimination of impurities or reduction of oxides (rGO), prevent re-oxidation, break C–O bonds, generate C–C bonds, and partially restore the sp^2^ hybridized –C– domains.

GO + ne^−^ + nILs^+/−^→ rGO (n−1) + nILs (7)

NC + ne^−^ + nILs^+/−^ → rNC (n−1) + nILs (8)

Herein, the high surface area of flat graphene nanosheets with ILs electrolyte generates additional active sites [163,172], thereby enhancing specific capacitance and energy storage [173]. Simultaneously, the high aspect ratio and hollow structure of NC facilitate ion transport [174], thereby providing efficient pathways for ILs electrolyte diffusion. Consequently, these factors contribute to an improved overall electrochemical performance [133,154], which is also influenced by the interconnected pore size distribution and directionality of the wall channel. In essence, a comprehensive understanding of the complexity of vdW interactions at the heterointerface can unlock the full potential of the quasi-2D/1D multilayer. Although this multilayer also consists of covalent and non-covalent interactions, the weak, yet profound, synergy of out-of-plane vdW interactions offers unprecedented control over the functionalities of the regenerated quasi-3D nanostructures. Therefore, the thoughtful implementation of vdW-driven functionalization has the potential to yield superior properties and proximity within well-defined quasi-2D/1D heterointerfaces.

## 4. vdW-Driven Transition

The prospect of transitioning from quasi-2D/1D graphene nanosheet and NC chain derivatives to advanced quasi-3D nanoarchitecture at the heterointerface unlocks a plethora of opportunities in numerous applications. This innovative integration generates a cutting-edge advanced functional material with enhanced attributes. These include superior mechanical properties, electrical and thermal conductivity, and sustainability derived from multi-constituents. In the ensuing subsections, we will delve into the potential of breakthroughs in transition driven by vdW forces within these heterointerfaces. Additionally, the significance of anisotropic quasi-2D intra–intersheet and quasi-1D NC intra–interchain configurations on the subsequent mechanisms that emerge from these interface transformations will also be explored. This is particularly in relation to in-plane and out-of-plane dynamics in load, charge, and heat transfer mechanisms.

### 4.1. Load Transfer (LT) Mechanism

**Figure 7:**
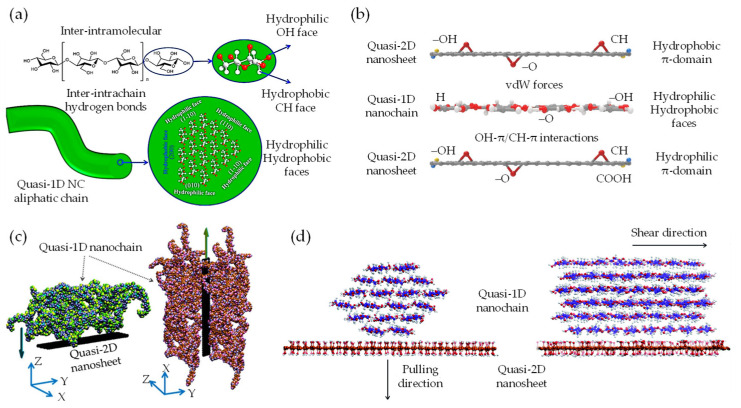
(**a**) Diagram showing anisotropic quasi-1D NC aliphatic inter–intrachain hydrogen bonding, featuring inter–intramolecular, along with hydrophilic–hydrophobic, crystalline faces (red: O, white: H, grey: C.) [47]. Reproduced with permission. Copyright: Elsevier B.V. (**b**) Depiction of hydrophilic–hydrophobic characteristics and vdW interactions within the quasi-2D/1D heterointerfaces (red: O, white/yellow: H, grey/blue: C). (**c**) Interface models (I and II) depicting quasi-2D/1D heterointerfaces under separation schemes in uniaxial and shear directions [94]. Reproduced with permission. Copyright: American Chemical Society. (**d**) Graph illustrating the normalized potential of mean force (PMF) in quasi-2D/1D heterointerfaces at various separation stages along the pulling and shear directions [95]. Reproduced with permission. Copyright: American Chemical Society.

The vdW-driven transition of quasi-2D/1D heterointerfaces towards quasi-3D nanoarchitecture is primarily governed by vdW forces [175,176,177]. This process involves a captivating interplay of forces, resulting in notable micromechanical properties. The transformation relies on a balanced interaction between cohesive-pullout bonded and non-bonded forces, specifically, the attractive–repulsive nature of vdW forces [94,95,178]. While cohesive-pullout forces ensure the structural integrity of the material and dictate its response to stress and strain, including fracture patterns [51], vdW forces play a significant role due to the proximity-dependent and orientation-dependent characteristics [92], despite being weaker than covalent or ionic bonds. These vdW forces drive the molecular organization within the heterointerface, facilitating the transition towards a quasi-3D nanoarchitecture [18,74]. Hence, understanding the load transfer (LT) mechanism between quasi-2D nanosheet and quasi-1D nanochain heavily relies on interfacial interactions at the heterointerface. Fundamentally, highly anisotropic quasi-1D NC [82,83,84], exhibiting ordered crystalline structures and strong inter–intrachain hydrogen bonds [81], possesses an impressive elastic modulus (150 GPa), similar to Kevlar [102]. The dense inter–intramolecular packing, as depicted in Figure 7a, augments the amphiphilic nature by forming hydrophilic (110/010) and hydrophobic (1$\bar{1}$0) facets through –OH and CH groups, respectively [179]. This arrangement promotes synergy and affinity with other inorganic amphiphilic nanomaterials, such as graphene derivatives in quasi-2D aromatic nanosheets [47,180]. These derivatives possess hydrophilic–hydrophobic π-domains, which interact through vdW forces [51,92,181], as illustrated in Figure 7b. Recent studies have emphasized the significance of vdW forces in the interaction of quasi-2D/1D heterointerfaces [79,92,94,95,96,97,98]. Through molecular-scale MD simulations, these studies have demonstrated the vital contribution of bonded and non-bonded vdW interactions at the heterointerface. Rahman et al. [94,101] explored the vdW interaction of quasi-2D monolayer and quasi-1D nanochain, maintaining an initial distance (<2 Å) through interface models (I and II) in a MD framework (Figure 7c). The study revealed that separating the quasi-2D nanosheet from the quasi-1D nanochain through normal and shear directions [182] resulted in reaction forces identified as F_cohesive_ and F_pullout_. Correspondingly, the in-plane Young’s modulus along the X/Y-axis (E_X_: 11.78 GPa and E_Y_: 32 GPa), which symbolizes quasi-2D/1D stiffness, is largely governed by the volume fraction, ranging from monolayer to trilayer. However, the out-of-plane Young’s modulus (E_Z_: 0.08–2.81 GPa), portraying quasi-2D/1D deformation along the Z-axis, is predominantly influenced by vdW interactions, as demonstrated in the non-bonded energy evolution (4650 kcal/mol) [101]. Mianehrow et al. [95] provide insight into the LT mechanism and the role of quasi-ID inter–intrachain in micromechanics, using potential of mean force (PMF) analysis. The study found that the F_cohesive_ and F_pullout_ between quasi-2D/1D (Figure 7d) at separation distances (≈1–3 nm) are markedly influenced by the orientation of hydrophilic–hydrophobic faces within and between inter–intrasheet and inter–intrachain. This influence results in substantial deformation, notably, without any observable failure at the interface. Likewise, Alqus et al. [79] have affirmed the crucial function of the quasi-ID NC amphiphilicity nature (CH and OH faces) configurations in maintaining inter–intrachain hydrogen bonds and the orientation of hydroxymethyl groups. Interestingly, in the presence of a polar solvent, the hydrophilic 010 faces of the quasi-1D NC chain tend to realign or reorient towards high-density CH-π interactions with the quasi-2D sheet, while simultaneously remaining shielded from the polar solvents.

**Figure 8:**
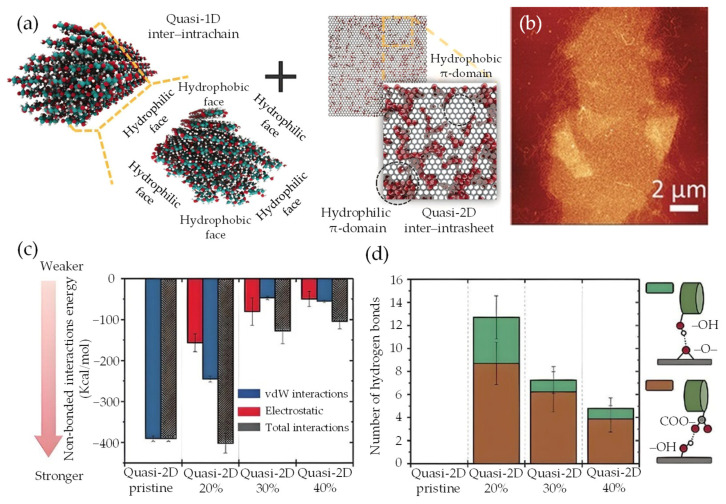
(**a**) Quasi-2D/1D assembly driven by amphiphilicity via hydrophilic–hydrophobic interfaces; (**b**) AFM image displays morphological interface interactions in quasi-2D/1D (approx. 20% nanosheet oxidation); (**c**,**d**) Bonded and non-bonded interfacial interactions at quasi-2D/1D interfaces with variable surface oxidation on quasi-2D nanosheets [92]. Reproduced with permission. Copyright: WILEY-VCH.

This reconfiguration restricts water intrusion into the hydrogen bonds between the quasi-1D inter–intrachain, thus underscoring the stability of the hydrophobic 100 faces and the amphiphilic nature of the system [47]. The rearrangement, which enhances the micromechanics of quasi-2D/1D interfaces, has empirically shown that enduring vdW interactions between the hydrophobic 1$\bar{1}$0 face and the π-electron mechanism serve to reinforce intrinsic structural integrity. Zhang et al. [99] consistently provide evidence supporting the significance of directional interfacial hydrogen bonds within an anisotropic quasi-1D NC chain, a concept depicted in Figure 7a. Inter–intrachain hydrogen bonds are notably recognized for their rapidly breaking and reform abilities [96]. Moreover, the findings offer valuable insights into the preferred vdW interactions within the quasi-2D/1D assembly, as shown in Figure 7b. In an experimental study, Xiong et al. [92] have made significant strides in clarifying the decisive influence of vdW interfacial interactions in hydrophobic–hydrophobic quasi-2D/1D heterointerfaces (Figure 8a) and the correlated micromechanical behaviors. Established findings were achieved by modulating the surface chemistry of the quasi-1D NC chain (Figure 8a-left), specifically, by incorporating anionic –COO– carboxyl groups onto its hydrophilic planes [183]. Synchronously, modification was made to the quasi-2D monolayer, recognized for its partially hydrophobic domains nature due to the inconsistently oxidized surface (Figure 8a-right). The distinct features of these highly oxidized amphiphilic GO nanosheets can primarily be attributed to the presence of –OH functional groups and epoxy bridges on the lateral planes, as well as the interaction of hydrophilic π-domains at edges or defects with –COOH carboxyl groups. Such alterations enriched the surface charge [90] at basal–edge domains (Figure 8a), as demonstrated by a zeta potential of −50 mV, bolstering the stability of vdW interfacial interactions. An intriguing observation was the drop-in zeta potential from −50 mV to −30 mV, which reduced repulsive Coulombic interactions, thereby maintaining the lateral integrity of overlap at the quasi-2D/1D sheet/rod-like-shape interfaces (Figure 8b) via hydrogen bonding and vdW interactions.

However, as affirmed by Xiong et al., MD simulations [92] show a tendency towards disassembly when oxidation levels exceed 20%. This is mainly attributed to reduced non-bonded interfacial energy and hydrogen bonding, as illustrated in Figure 8c,d. These findings emphasize the critical role of non-bonded interfacial interactions, especially vdW forces, in driving the transition of quasi-2D/1D heterointerfaces via adsorption and hydrogen bond formation. Notably, this insight aligns with prior studies [184] that associated weaker interfacial bonded and non-bonded interactions with higher oxidation states. These interfacial interactions and synergistic crosslinking lead to the alignment of ultra-strong, yet flexible, quasi-2D/1D interfaces with an impressive elastic modulus (182 ± 56 GPa), enabling the construction of robust and porous laminated nanoarchitecture with rapid load transfer capabilities [89,90,92,156,185,186]. These approaches pave the way for creating novel hierarchical quasi-3D nanoarchitecture that capitalizes on the apt amphiphilic nature and synthetic constituents, along with the transformation of the interfacial interactions and micromechanical behavior.

### 4.2. Charge Transfer (CT) Mechanism

**Figure 9:**
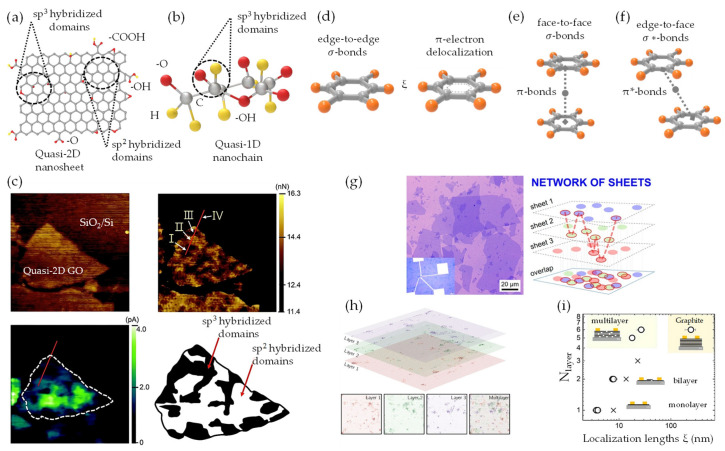
(**a**) An atomically flat quasi-2D graphene nanosheet derivative, illustrating pristine sp^2^ alongside tetrahedral sp^3^ hybridized domains; (**b**) Quasi-1D NC chain sp^3^ hybridized domains; (**c**) Topographical images and sp^2^/sp^3^ regions map (1.5 × 1.5 μm^2^) of quasi-2D nanosheet (~0.8 nm) on SiO_2_/n-Si substrate [57]. Reproduced with permission. Copyright: The Royal Society of Chemistry. (**d**–**f**) Hexagonal lattice and tetrahedral geometry configuration of quasi-2D depend on σ/π-bonds, charge localization length (ξ), and π-electron delocalization in edge-to-edge, face-to-face, and edge-to-face, respectively; (**g**) Top and lateral views of quasi-2D trilayer model, illustrating ξ dependence on the number of layers (N_layer_) and showing scale-independent sp^2^ hybridized domains (colored circles) separated by border defects, and a random path across overlapping (dashed red lines) [91]. Reproduced with permission. Copyright: American Chemical Society. (**h**) Schematic of localized states (ψ_A_, ψ_B_, ψ_C_) and local densities (red, green, blue) quasi-2D mono–bi–trilayer (top and bottom); (**i**) Dependence of ξ on N_layer_ in quasi-2D mono–bi–trilayer; circles denote experimental data, crosses indicate simulation results [100]. Reproduced with permission. Copyright: American Chemical Society.

The transition occurring within the reconstituted quasi-3D nanoarchitecture is primarily driven by vdW forces. These forces facilitate the assembly and functionalization of quasi-2D inter–intrasheet and quasi-1D inter–intrachain. This intricate process encompasses the restoration of the pristine electrical conductivity, along with associated inherent electronic characteristics. Accordingly, a combination of unaltered graphene-like sp^2^ and interconnected functionalized sp^3^ hybridized domains emerges, enabling the nanoarchitecture to shift from an insulator to a semiconductor and, ultimately, to a graphene-like semimetal [59]. The transition depends on the ordered and disordered degree of the structural configurations [187]. Fundamentally, as demonstrated in Figure 9a, the quasi-2D GO/rGO sp^2^ hybridized domains are regions where C atoms are bonded to three other atoms in a planar geometry forming a hexagonal lattice, while sp^3^ hybridized domains occur when C atoms are bonded to four other H/O atoms in a tetrahedral geometry [57]. In quasi-1D NC, sp^3^ hybridized domains (Figure 9b) represent areas where C atoms maintain tetrahedral geometry, resulting in a stable, ordered crystalline structure [188]. The presence of the –OH group in these regions enables hydrogen bonding, which is vital for superior mechanical properties, i.e., strength, flexibility, stiffness, and stability. Lee et al. [57] investigated monolayer quasi-2D GO structure containing sp^2^/sp^3^ regions using conductive AFM for local friction and conductance characterization (Figure 9c). The study findings revealed that this quasi-2D nanosheet (Figure 9c-right top corner) exhibited sp^2^/sp^3^ regions between 10 and 100 nm in size, with low friction and high conductance in sp^2^-rich phase (I and III) and high friction and low conductance in sp^3^-rich phase (II). Additionally, current voltage spectroscopy (Figure 9c-left bottom corner) demonstrated the influence of –O, –OH, –COOH and epoxy bridges on local current flow in monolayer quasi-2D GO (white dashed and red line) varying sp^2^/sp^3^ carbon ratios in the domains and subdomains. These results highlight the importance of spatial mapping for rapidly identifying heterogeneous composition at the nanoscale, which is crucial for understanding the charge transfer (CT) mechanism. Figure 9d notably demonstrates that in atomically flat pristine graphene, characterized by sp^2^ hybridized domains within the localization length (ξ), CT predominantly occurs via strong σ-bonds, weaker π-bonds, and π-electron delocalization within the hexagonal lattice [52]. In the quasi-2D mono–bilayer configuration that holds sp^2^/sp^3^ regions, the π-electrons delocalized on the sp^2^ hybridized domains largely determine the electronic structure [58]. This is because the π/π* states lie within the σ/σ* states (Figure 9e), which originate from the sp^3^ hybridized domains [57,104,189]. More significantly, the orientation of quasi-2D mono–bilayer structures, particularly, the shift from isotropic to anisotropic alignment, considerably influences the electrical properties. Herein, it is important to note that the in-plane and out-of-plane CT mechanisms within these configurations has been infrequently investigated. In edge-to-edge configurations (Figure 9d), in-plane CT is primarily governed by sp^2^ C–C σ-bonds through the variable-range hopping (VRH) mechanism [103,104] between localized states. However, the strong covalent nature of σ-bonds hinders electron mobility, resulting in less efficient CT [190]. In accordance, the out-of-plane CT within the face-to-face configuration, as depicted in (Figure 9e), is mainly controlled by π-conjugated ξ regions via the sp^2^ hybridized domains overlapping mechanism [100] at the interfaces of nanosheets. Consequently, this allows electrons to traverse π-bonds more easily, rendering the out-of-plane CT mechanism more effective [100]. In an intriguing observation, two adjacent nanosheets in an edge-to-face configuration (Figure 9f) exhibit distinct CT components involving both σ*/π*-bonds via hopping and overlapping mechanisms. Notably, enhanced out-of-plane CT is facilitated by stronger π-electron delocalization at the edge interface through π*-bonds. However, the presence of σ*-bonds within the sheet layer hinders in-plane CT compared to face-to-face or edge-to-edge configurations. Thus, the CT in the edge-to-face orientation is predominantly out-of-plane, rendering the in-plane component statistically insignificant or solely associated with a single sheet [91]. Recent studies have delved into the experimental and computational investigations of quasi-2D mono–bilayer [57,91,93] and multilayer stacks [100] within a unified CT domain. Experimentally, the degree of oxidation (sp^2^/sp^3^ regions), interlayer spacing (*d*, d_0_, d_vdW_), lateral dimensions (in-plane/our-of-plane), and thickness (N_layer_) serve as independent parameters that define the CT mechanism and its associated properties. Kovtun et al. [91] proposed a scale-independent model (Figure 9g) to investigate CT in disordered quasi-2D networks. This model allows the size of the sp^2^ hybridized domain to be neglected because ξ significantly surpasses the average sp^2^ region size. Instead, the model emphasizes the interlayer distance (*d*) between overlapping aromatic regions (ϕ) to derive a generalized expression outlining ξ dependence on N_layer_ as the following:

(9) $$\xi \left(N_{layer}\right)\sim \frac{\phi }{2}+d.\sum_{i}^{N_{\mathrm{layer}}}n\left(i\right)$$

where *n(i)* denotes the number of steps or conductive sp^2^ regions involved in the stack. Remarkably, in a regenerated quasi-3D film [91], in situ reduction layers lead to a decrease in the number of steps (*i*) and thickness (nm). These parameters approach a critical value (N_layer_ ≈ 8), where the continuous percolated random path disintegrates into smaller connected regions (Figure 9g), leading to a significant decline in ξ. Ultimately, in a single-sheet film (N_layer_ = 3), non-overlapping sp^2^ regions (red circles) result in ξ approximating twice the typical sp^2^ domain size (orange circles) in quasi-2D (<10 nm).

**Figure 10:**
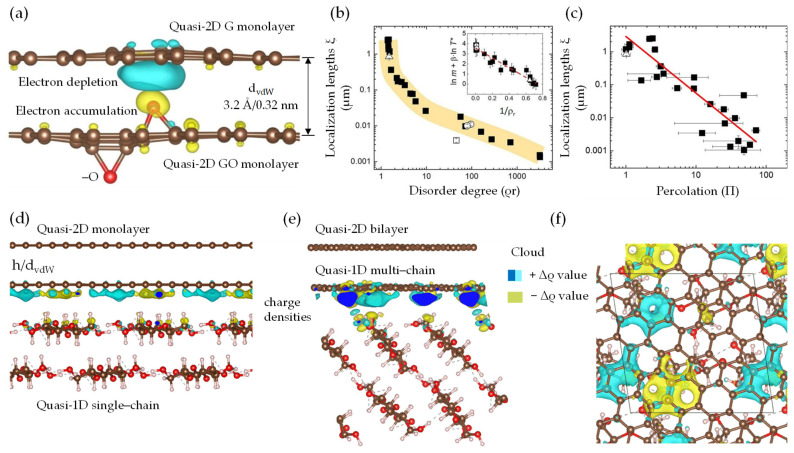
Geometric phase transition for single- and multiscale CT regimes in quasi-2D/1D heterointerfaces representation. (**a**) Charge density variations in quasi-2D G/GO monolayer heterointerfaces from DFT simulation: C atoms (brown), O atoms (red), electron accumulation (yellow), and depletion (blue), respectively [93]. Reproduced with permission. Copyright: Springer Nature. (**b**,**c**) Localization length ξ vs. percolation (Π) and degree of disorder (ρr) description of the CT in quasi-2D rGO networks in term of metal−insulator phase transition [91]. Reproduced with permission. Copyright: American Chemical Society. (**d**–**f**) Schematic view of quasi-2D/1D mono–bilayer and single–multichain iso-surfaces/interface region showing localized differential charge densities and net charge transfer (Δρ), with positive values in blue and negative values in yellow [51]. Reproduced with permission. Copyright: Royal Society of Chemistry.

Computationally, the MD simulation of CT properties in networked structures, as opposed to individual sheets, necessitates the consideration of inter–intrasheet interactions, multiscale processes, and the inherent diminished quality of sheets exhibiting defects, chemical functionalization, and size polydispersity [51,91,100]. Utilizing a multiscale computational method, Çınar et al. [100] have firstly reported a multiscale model of disordered vdW quasi-2D trilayer to elucidate the CT mechanism, considering the interlayer interaction role and various types of defects (i.e., Pentagon). In the devised quasi-2D multilayer model (Figure 9h), featuring a rectangular ribbon-like geometry that is 20 nm wide with diverse disorder distribution (i.e., epoxides), an intriguing transition from Efros−Shklovskii (ES-VRH) to a partially overlapping CT mechanism is observed as N_layer_ amplifies. This amplification accentuates a dominant impact of the bulk material on the CT properties [100]. Notably, within the VRH framework, the correlation between N_layer_ (2 ≤ N_layer_ ≤ 6) and ξ (monolayer: 7.8 nm, bilayer: 13.7 nm, and trilayer: 22.9 nm) in quasi-2D assemblies is elucidated (Figure 9i). Overlapping layers with localized states (ψ_A_/ψ_B_) promote increased transmission by allowing more probable hopping among energetically similar and spatially distinct states through intermediate states (ψ_C_). Still, a comprehensive framework to elucidate CT in multiscale regimes across multiple vdW heterointerfaces remains elusive. In light of this progress, we will explore the CT mechanism from two distinct perspectives: the single-scale and the multiscale CT regimes. In the single-scale CT regime mechanism, a prototypical building block constituted of a quasi-2D mono–bilayer is employed, which features semi-conductive graphene lattice interspersed with oxygen-related defects. For random quasi-2D monolayer networks, the nearest-neighbors hopping (NNH) model prevails owing to the presence of localized states (ψ) at a characteristic distance (ξ) from disrupted defects and functional groups within the hexagonal lattice. Regarding disorder and metal−insulator phase transition (MIT) [187], percolation is a critical concept in understanding the CT mechanism. In CT for quasi-2D mono–bilayer networks, percolation occurs as the material shifts from insulating to conducting, with increased conductive pathway density. This can be achieved by in situ reduction approaches [29,159,162,191,192], which reduce oxygen functionalities, increase sp^2^ –C– domains, and restore conductivity [161,165,167]. When conductive sp^2^ –C– region density increases, interconnected pathways form, facilitating charge carrier flow and reaching a percolation threshold. In a condensed version, Haidari et al. [93] employed DFT simulations to reveal charge density differences in vertically stacked quasi-2D G/GO monolayer heterointerfaces. The insights gained shed light on the impact of interlayer coupling on the CT mechanism, as illustrated in Figure 10a. Importantly, they also found no chemical bonding in the quasi-2D monolayer, as confirmed by DFT calculations of CT characteristics. The possibility of electron flow possible from the quasi-2D monolayer arises due to varying charge density, represented by electron depletion (blue) and accumulation (yellow) areas [193]. This phenomenon is attributed to oxygen groups (red) in quasi-2D GO, induced by weak vdW interactions at an interlayer distance (d_vdW_) of 3.2 Å/0.32 nm. As a proof of concept, Sadasivuni et al. [29] have simplified the proximity-sensing mechanism in quasi-2D/1D multilayer (N_layer_ ≈ 40) to the efficient CT and electric field distribution. In equilibrium scenarios associating quasi-2D/1D multilayer (N_layer_) heterointerfaces, in-plane covalent interactions may yield high reproducibility and uniform electrostatic fields with charge. Concurrently, weak out-of-plane vdW non-covalent interactions could stimulate doping between the multilayer without necessitating chemical bonding. This process may enhance CT due to fluctuations in charge density and accumulation or depletion of electrons. It is noteworthy to underline that charge carriers can traverse insulating barriers in quasi-2D GO/rGO via fluctuation induced tunneling (FIT) [194] across sp^3^ hybridized regions. Similarly, tunneling probability increases as the size of sp^3^ regions decreases or the energy barrier lowers, thereby boosting CT in quasi-2D GO/rGO mono–bilayer networks. As the localization length (ξ) exhibits asymptotic growth, the degree of disorder (ρ) diminishes correspondingly. This negative linear correlation (Figure 10b) is evidenced in semiconducting π-conjugated quasi-1D filler [91], akin to a spaghetti-like structure. The percolation model is dominated (Figure 10c), facilitated by a 3D network of interconnected isolated sp^2^ regions, which allows charge carriers to traverse between the two layers. Consequently, the existence of a 3D network permits extended π-conjugation and delocalized π-electrons, which results in high electrical conductivity. As comprehensively discussed by Kovtun et al. [91], quasi-conductive fillers form a complex framework akin to a spaghetti-like configuration, wherein the electrical conductivity is directly proportional to the aggregate length (ξ). This study emphasizes the relationship between complex networks of disordered quasi-2D nanosheets and quasi-1D conductive channels [195]. It elucidates that CT in low-dimensional systems adheres to the ES-VRH model. This assertion is backed by the works of Epstein et al. [196] and Fogler et al. [197], as these systems evolve into a quasi-3D network composed of numerous quasi-2D/1D contact regions. Nevertheless, in the quasi-3D nanoarchitecture, the CT dynamics are governed by a multiscale CT regime, which encompasses quasi-2D G/GO/rGO nanosheets as primary pathways and quasi-1D NC chain for structural reinforcement. The CT mechanism integrates isolated sp^2^ and interconnected sp^3^ hybridized domains, employing hopping and overlapping mechanisms within the nanoarchitecture. The interlayer among neighboring quasi-2D/2D nanosheets is facilitated by the hopping and tunneling CT mechanism [198] across the peripheral or interpenetrating regions.

The synergistic influence of vdW forces and π-π interactions between the layers expedites this phenomenon. Furthermore, functional groups, such as –O, –OH, and –COOH, serve as CT sites, enabling the establishment of hydrogen bonds and thus promoting efficient CT pathways throughout the nanoarchitecture framework. To further clarify the CT mechanism in quasi-3D nanoarchitecture based on insulator and semimetal interfaces, we delve into the roles of π-electron delocalization and π-conjugated regions. Additionally, Silvestre et al. [51] analyzed the differences in charge density induced by ex situ electric fields and mechanical strains. Figure 10d–f provides quasi-2D/1D structural models, elucidating the vdW interactions between quasi-2D mono–bilayer and hydrophilic–hydrophobic (001/110) faces of quasi-1D NC single–multichain. The charge accumulation maps (Figure 10d–f) within these quasi-2D/1D heterointerfaces display an inhomogeneous net charge distribution (Δρ) across the quasi-2D layer, resulting from varying orbital hopping magnitudes between the quasi-1D NC iso-surface and the quasi-2D π orbitals. This CT predominantly occurs at the quasi-2D/1D heterointerface, as evidenced by the negligible charge density variation (Δρ ≈ 0) within the iso-surfaces and interface. The observed net charge localization, with a Δρ of approximately 0.2 × 10^13^ e cm^−2^, suggests the formation of discrete electronic transmission channels at the quasi-2D/1D heterointerface. However, it is crucial to acknowledge that such an inhomogeneous Δρ, which generates regions enriched with electrons and holes in the quasi-2D layer, could detrimentally impact the electronic transport properties. This work has paved innovative pathways for implementing vdW-driven transition, enabling the strategic rearrangement of nanosheet and nanochain configurations into macroscopic nanoarchitecture. Yet, a comprehensive understanding of the underlying principles governing these vdW heterointerfaces and the CT mechanisms responsible for the superconducting tendencies remains to be elucidated. Therefore, further clarifying these sophisticated interplays will shed more light on the CT mechanisms within the quasi-2D/1D graphene inter–intrasheet and NC inter–intrachain matrices.

### 4.3. Heat Transfer (HT) Mechanism

**Figure 11:**
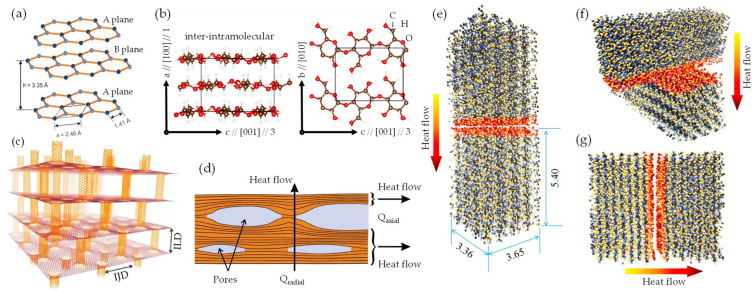
(**a**) Schematic depiction of the atomic configuration in anisotropic graphene monolayer [112]. Reproduced with permission. Copyright: Springer Nature. (**b**) Schematic representation of anisotropic quasi-1D NC rod-like inter–intramolecular configuration: aligned cellulose chains Iβ along the a-axis with weaker bonding along the b-axis compared to c-axis direction [202]. Reproduced with permission. Copyright: IOP Publishing. (**c**) Schematic representation of quasi-3D nanoarchitecture integrating carbon nanotube pillars and graphene sheets, enabling adjustable λ_X_ and λ_Z_ [112]. Reproduced with permission. Copyright: Springer Nature. (**d**) Schematic depiction of anisotropic (Q_axial_/Q_radial_) thermal conductivity in quasi-2D graphene multilayer [161]. Reproduced with permission. Copyright: WILEY-VCH. Atomistic models with interface regions (highlighted in red) used to evaluate thermal resistance: (**e**) chain, (**f**) transverse perpendicular, and (**g**) transverse parallel configurations [145]. Reproduced with permission. Copyright: American Chemical Society.

Exploring the thermal dynamics within a quasi-3D nanoarchitecture, synthesized through the vdW-driven assembly of flat quasi-2D GO/rGO nanosheet and hollow porous quasi-1D NC chain, reveals a complex interplay of forces and structural dimensions. This interplay not only refines our understanding of the efficient heat transfer (HT) mechanism, but also underlines the role of nanoarchitecture design in modulating effective thermal conductivity (λ_eff_). As such, it provides a framework for tailored thermal management in the development of next-generation advanced functional materials. Herein, the terms in-plane and out-of-plane thermal conductivity [39,105,106] define the directional differences (axial and radial) in HT within thermal interface materials [199]. In-plane thermal conductivity (λ_X_) refers to HT along the plane (Q_axial_), while out-of-plane thermal conductivity (λ_Z_) represents HT perpendicular to the plane (Q_radial_). Due to the specific structural configuration, different materials display distinct thermal conductivities in these two directions. This disparity results in an observable effect known as anisotropic (λ*_X_*/λ*_Z_* or Q_axial_/Q_radial_) thermal conduction behavior [105,106,109]. When it comes to the high-quality anisotropic quasi-2D graphene monolayer (Figure 11a), it has an extremely high λ_X_ (2000–5000 mW m^–1^ K^–1^) [107] in a vacuum, primarily due to covalent sp^2^ bonding of C atoms allowing rapid phonon (heat carrier) transfer [200]. However, its λ_Z_ heat flow (≈ 20 mW m^–1^ K^–1^ at 298 K) is restricted by non-covalent weak vdW interactions [112,201]. Similarly, the anisotropic quasi-1D NC rod-like chain exhibits considerable λ_X_ (≈900 mW m^–1^ K^–1^) and λ_Z_ (240 ≈ 520 mW m^–1^ K^–1^ at 298 K) [202]. These features are attributed to its unique inter–intramolecular configuration and inter–intrachain orientation of polymerized units (Figure 11b), precisely, cellulose Iβ [108]. Inherently, covalent bonds facilitate HT, whereas weaker vdW bonds impede the propagation of phonons [202]. Nonetheless, this significance lies under intrinsic thermal properties at a highly ordered degree status. Recent research suggests that the transition into quasi-3D nanoarchitecture [112,203] can mitigate adverse thermal effects, largely due to the specific role of weak vdW forces in multilayer assemblies. For instance, quasi-1D carbon nanotubes (CNTs), as pillared within quasi-2D graphene nanosheets, along with other similar formations, are paving the way for the future of nanoarchitecture by offering versatile thermomechanical functionality [203]. In terms of HT, research indicates that the thermal properties of this quasi-3D nanoarchitecture heavily depend on lateral quasi-1D separation and the interlayer distance between graphene sheets [112]. Also, manipulating the interjunction distance (IJD) and interlayer distance (ILD) can mitigate the inadequate interlayer thermal coupling observed in a stack of graphene sheets (Figure 11c). Subsequently, a longer IJD and shorter ILD would reduce the λ_Z_, making it more suitable for thermal insulation or thermoelectric purposes [112]. The effectiveness of these innovative structures lies in the fusion of quasi-2D and quasi-1D interfaces and characteristics, making them applicable for diverse uses as cutting-edge thermal transport materials.

HT in quasi-3D nanoarchitecture [19,24] is typically a complex process that can involve conduction (transfer of heat by direct contact), convection (transfer of heat by movement of a fluid), and radiation (transfer of heat via waves). Fourier’s Law defines the conductive heat flux Q through porous structure [204] as the product of its thermal conductivity λ, area A, and temperature gradient ($\frac{dT}{dx}$), represented by Equation (10):

(10) $$Q=-\;\lambda \;A\frac{dT}{dx}$$

Primarily, this largely pertains to nonconductive HT processes combined with the effects of solid/gas phase thermal conduction ($\lambda_{cond}^{s}/\lambda_{cond}^{g}$), thermal convection (λ*_conv_*), and radiative (λ_rad_) heat transfer phenomena in axial and radial directions (Equation (11)) within the aligned wall cell and pore channels of a porous structure [19,108,112].

(11) $$\lambda_{eff}=\lambda_{cond}^{s}+\lambda_{cond}^{g}+\lambda_{conv}+\lambda_{rad}$$

where $\lambda_{eff}$ is effective thermal conductivity. Notably, recent research by Apostolopoulou et al. [108] explored the λ and HT fundamental aspects of anisotropic quasi-1D NC rod-like chains in aligned anisotropic porous architectures as thermally insulating materials (i.e., foam). The term anisotropic (λ_axial/radial_) originates from the inherently high anisotropy of quasi-1D NC (λ_axial_/λ_radial_: 1.5–8.5) [108,109,110,111]. When combined with the unique rod-like alignment, this anisotropy leads to a markedly anisotropic distribution and density of internal interfaces in materials equipped with aligned graphene derivatives (Figure 11d). This property is useful in thermal management applications, where heat flow needs to be directed or restricted in certain directions [24,39]. Computationally, Diaz et al. [145], through MD simulations, have predicted HT at interface regions across different model configurations ranging from single crystals to organized nanoarchitecture. The findings revealed a significant correlation between λ and the alignment of quasi-1D NC rod-like structures along the heat flow direction (Figure 11e–g). When combined with these alignment configurations, the lowest thermal resistance detected for quasi-1D NC (9.4 to 12.6 m^2^ K GW^–1^) suggests unique prospects for managing thermal conductivity λ.

**Figure 12:**
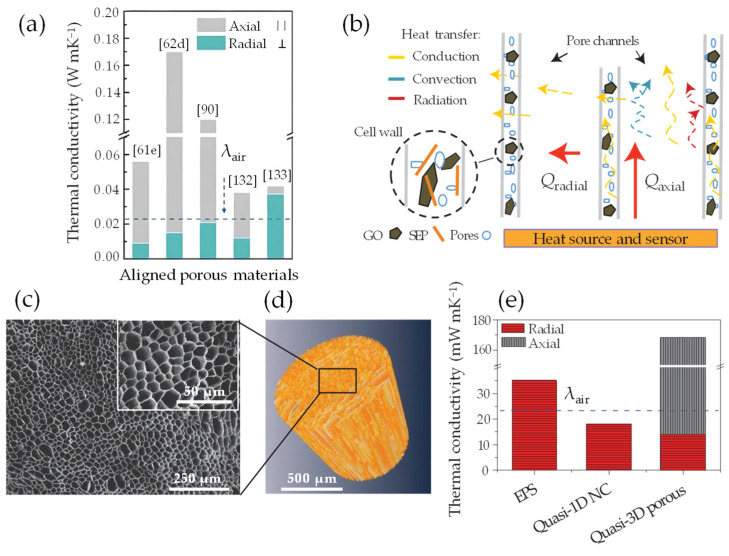
(**a**) Thermal conductivity values of conventional aligned porous materials in axial and radial directions [19]. Modified with permission. Copyright: WILEY-VCH. (**b**) Schematic view depicting the functions of conduction, convection, and radiation in managing heat flow within a quasi-3D nanoporous foam with directed mesoporous and relative free pathways in Q_axial_/Q_radial_ directions; (**c**,**d**) Morphological and microtomographic images illustrate the pore channels and cell walls of the reconstructed quasi-3D porous nanoarchitecture; (**e**) Thermal conductivity values of the quasi-3D porous foam in the Q_axial_/Q_radial_ directions compared with quasi-1D NC and conventional insulator polystyrene (EPS) [24]. Reproduced with permission. Copyright: 2015 Springer Nature.

The advantageous characteristics of the quasi-1D NC anisotropic structure, namely, its low density and high porosity [109,110,111], have been verified to contribute significantly to superior thermal insulation capabilities. Interestingly, a highly porous nanoarchitecture characterized by nanoscale pore size and cell wall channels (~50 μm) can significantly diminish overall thermal transmission and boost thermal insulation [109]. The restricted λ_conv_ and reduced λ_cond_ are largely facilitated by the quasi-3D fibrous–porous structure [205]. Such reduction is due to the relatively free path of gas emanating from mesopores alongside the multiple reflective λ_rad_. It is crucial to acknowledge that conventional thermal insulators, such as expanded polystyrene (30–40 mW m^–1^ K^–1^), possess low λ and excellent heat resistance [206]. Despite these gains, their use in practical applications is restricted due to less optimal mechanical properties, specifically, fragility, safety, and environmental concerns [206]. A need arises to lower λ below the air value (λ_air_: 25 mW m^–1^ K^–1^) to decrease the necessary space and insulation materials (Figure 12a). However, achieving super-insulating by replacing air with gas or vacuum [206] or manipulating pore size and morphology [207], while maintaining the free air path, remains challenging. Compared to conventional thermal insulators (Figure 12a), porous nanoarchitecture with pore structures assembled from bottom–up vdW heterointerfaces exhibit substantial anisotropic thermal insulation [19]. This feature is particularly beneficial for achieving well-structured cell and pore alignments with exceptional mechanical characteristics. Significantly, anisotropic quasi-3D nanoarchitecture-like films [39] and aerogels/foams [24] assembled from quasi-2D GO/rGO nanosheet and quasi-1D NC chain exhibit excellent thermal insulation. They demonstrate superior in-plane λ_X_ and inferior out-of-plane λ_Z_ thermal conductivities, coupled with desirable features, such as ultralight weight (7.5 kg m^–3^), robustness, and high porosity (99.5%). Accordingly, when considering the negligible interfacial HT, along with the in−plane and out−plane properties of the internal structure, it becomes evident that the anisotropic behavior (λ*_X_*/λ*_Z_* or Q_axial_/Q_radial_ > 1, indicating anisotropic) of quasi-2D/1D N_layer_ alignment has a significant impact on the overall HT (λ_conv_/ λ_cond_/λ_conv_) [24]. As comprehensively discussed by Wicklein et al. [24], the contributions of convection (λ_conv_), gas–solid conduction (λ_gas_–λ_solid_), and radiation (λ_rad_) have influenced the thermal transport properties within the oriented pore channels and cell wall of the quasi-3D porous foam (Figure 12b–d). Consequently, the λ_radial_ value of 15 mW m^–1^ K^–1^ is considerably below the λ_air_ and half that of conventional insulators (Figure 12e). In such a super-insulating quasi-3D porous foam, the nanosized constituents contribute to interfacial thermal resistance, illustrating the efficient thermal insulation with a homogeneous temperature distribution [24]. Furthermore, the minimization of thermal contact resistance and forming efficient, consecutive, thermally conductive pathways in the direction of heat flow are ascribed to the anisotropic quasi-1D NC hierarchical structures [39,88,106,109]. The vdW-driven transition within quasi-2D nanosheets and quasi-1D NC chains indeed holds the potential to stimulate the generation of highly efficient 3D thermally conductive pathways. Within this framework, the pursuit of super-insulating strategies that leverage ordered, aligned, quasi-3D porous nanoarchitectures, inspired by renewable hierarchies, could promote the advancement of high-performance thermal insulator materials. These innovative functional materials would not only possess energy efficiency, but also offer eco-effectiveness and cost-effectiveness merits.

## 5. vdW-Driven Alignment

The systematic arrangement of quasi-2D/1D building blocks is of critical importance, especially for large-scale applications, owing to the unique size and shape-dependent electronic properties they possess. Numerous methodologies have been devised to align these vdW heterointerfaces nanoarchitecture, among which the bottom–up strategies have proven highly effective. The Langmuir–Blodgett (LB) [78,114,115,131,208] assembly technique, for instance, leverages specific surface pressure conditions and dynamic inter–intramolecular forces during phase transitions at the air–liquid interface, thereby promoting interaction between quasi-1D/2D vdW heterointerfaces. Moreover, the versatile freeze-casting or ice-templating [19] technique has been employed to initiate in situ and ex situ freeze alignment induced patterns, allowing quasi-1D/2D vdW heterointerfaces to assemble into asymmetric, centrosymmetric, and symmetric structure patterns. Regarding interfacial interactions, these techniques provide a systematic and controlled strategy for constructing layered structures from the bottom up, incorporating both in situ and ex situ alignments. The subsequent subsections will delve into the alignment induced routes at the liquid–solid interface and explore the potential in the single- and dual-aligned frameworks of quasi-1D/2D vdW heterointerfaces.

### 5.1. In Situ Alignment Induced

**Figure 13:**
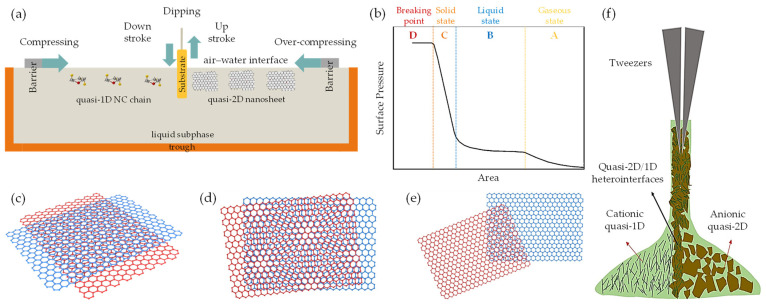
(**a**) Schematic view of in situ alignment induced quasi-2D/1D heterointerfaces through Langmuir–Blodgett (LB) assembly technique; (**b**) Surface-pressure isotherm graph illustrating the phase transition during compression and over-compression; (**c**–**e**) Schematic view demonstrates the face-to-face and face-to-edge configurations of quasi-2D/2D mono–bilayer; (**f**) Schematic representation of the in situ alignment process of quasi-2D/1D heterointerfaces into continuous filament, induced by charge surface manipulating [90]. Reproduced with permission. Copyright: Elsevier B.V.

Built on the surface pressure-area isotherm (π-A isotherm), which functions for each floating monodispersed layer, a typical transfer process into solid substrates is governed by Langmuir compression at the air–liquid interface [115,208]. When the quasi-2D or quasi-1D monolayer is compressed isothermally (Figure 13a), it transitions from a gaseous state to a liquid state and then to a solid state [209]. This significant change in the surface-pressure isotherm graph (Figure 13b) induces high monolayer density due to the inter–intramolecular forces [209]. Consequently, the nature of the surface charge and geometric configurations (Figure 13c–e) between the mono–bilayer is crucial for molecular rearrangement and alignment on hydrophilic–hydrophobic substrates at the liquid subphase. At a certain collapse pressure state beyond the solid state (stage C), the phase transition reaches the horizontal breaking point (stage D) extrapolated from the isotherm graph (Figure 13b). This collapse upon over-compressing generates an intriguing phenomenon where the quasi-2D monolayer assembles into a quasi-3D multilayer nanoarchitecture. Notably, Jaafar et al. [114] have employed the LB technique with an unconventional dipping and collapse pressure (>15 mN/m) protocol to regenerate highly porous 3D surface topography from a quasi-2D rGO monolayer. This work has paved novel routes for assembling quasi-2D smooth surfaces into 3D porous cavities and interconnected pores through in situ alignment induced by over-compressing. In light of this, modified in situ liquid subphases and setups, including barrier-free, distance, and surface spreading pressure, permits the dip-coating of large-area substrates and facilitates continuous roll-to-roll deposition [113]. However, there is a necessity to control face-to-face and face-to-edge geometric configurations (Figure 13c–e) thru in situ manipulating monodispersed colloids. Accordingly, in situ reduction strategies could eliminate unnecessary surface functional groups, a critical step in controlling face–edge functional groups.

**Figure 14:**
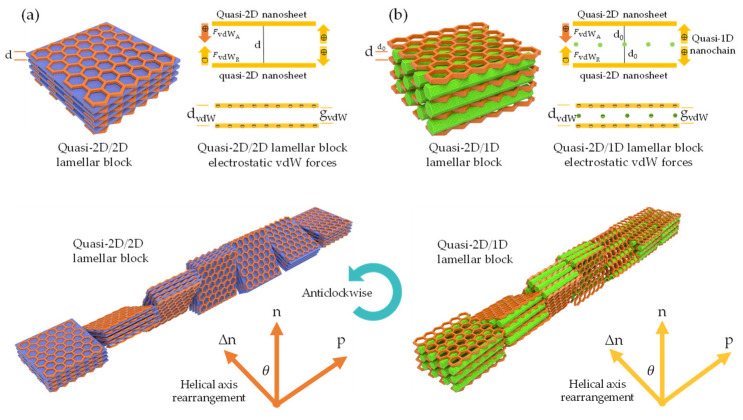
Templating kinetics and driving forces of (**a**) quasi-2D/2D and (**b**) quasi-2D/1D heterointerfaces into specific rearrangement, incorporating helical pitch (p), interlayer distance (*d*, d_0_, dv_dW_, gv_dW_), and anticlockwise twist/rotation (∆n, n vector, θ angle) (surface functional groups have been omitted for clarity) [125]. Modified with permission. Copyright: Nature.

As previously discussed in Section 2, it should be noted that when quasi-2D/1D heterointerfaces interact with each other, they experience vdW attraction and electrostatic repulsion due to multiple non-covalent interactions on the faces and edges. Additionally, the electrostatic repulsion in an anionic NC polar environment can enhance graphene derivatives dispersion and reduction [89]. Moreover, the surface charge modifications via multifunctional ILs^+/–^ [89] or organic coagulants [210] lead to extra electrostatic stabilization. This in situ process allows colloids to gain more effective gluing capabilities through non-covalent interactions, yet maintaining strong covalent interactions. In the study by Zhang et al. [90], empirical evidence demonstrates the effectiveness of surface charge manipulation, either anionic or cationic, applying in situ reduction strategies in quasi-2D/1D monodispersed colloids (Figure 13f). The researchers accomplished in situ alignment of conductive filaments (σ_filament_: 3298 ± 167 S/m) via the interfacial nanoparticle complexation [211] and charge neutralization that exists between anionic (rich in –COO groups) quasi-2D GO nanosheets and cationic (rich in –NH^3+^ groups) quasi-1D NC chain interfacial interactions. A lamellar block model of quasi-2D/2D and quasi-2D/1D vdW heterointerfaces is proposed (Figure 14a, b), building upon anisotropic helical properties inherent in quasi-2D graphene nanosheets [124,125,126] and quasi-1D NC chain [5,122,123,126,145,212]. Such hetero-interfaces aligned parallel and perpendicular into a helical order along a helical axis (Figure 14a,b-top right corner), identified by the ∆n/ns vector (Figure 14a,b-bottom right corner), and are marked by adjacent interlayer spacing parameters (d, d_0_, d_vdW_, g_vdW_). The inherent polarity within the charge density nature at the boundaries, surfaces, and interfaces promotes a magnet-like effect, whereby adjacent blocks may either attract or repel each other (Figure 14a,b-bottom).

Delving deeper, the free energy minimization mechanism found in LCs colloids [213] drives the formation of rotated or twisted conformations, either clockwise or anticlockwise, at a distinct angle (θ). This further emphasizes the dominant role of electrostatic vdW forces in governing helical rearrangements during liquid–solid phase transitions along the helical axis [125]. Consequently, potential enhancement in the mechanics and optics (i.e., helical pitch) of the regenerated quasi-2D/1D nanoarchitecture could be achieved [126,214]. Such insights emerge from novel design concepts for mechanochromic, thermochromic, and colorimetric sensors responding to external stimuli [126,214]. Concerning in situ alignment of chiral nematic LCs colloids [215] via vdW forces, nano-entities are aligned prior to being dispersed within the LCs structure [121,123,124,125,126]. This can be facilitated using either surface with specific textures [123] or patterns as templates [121] to assist in achieving the desired orientation [121,127]. These findings have not only enriched our understanding of the in situ alignment induced in quasi-2D/1D heterointerfaces, but also provided innovative designs and functionalities in macroscopic nanoarchitecture.

### 5.2. Ex Situ Alignment Induced

**Figure 15:**
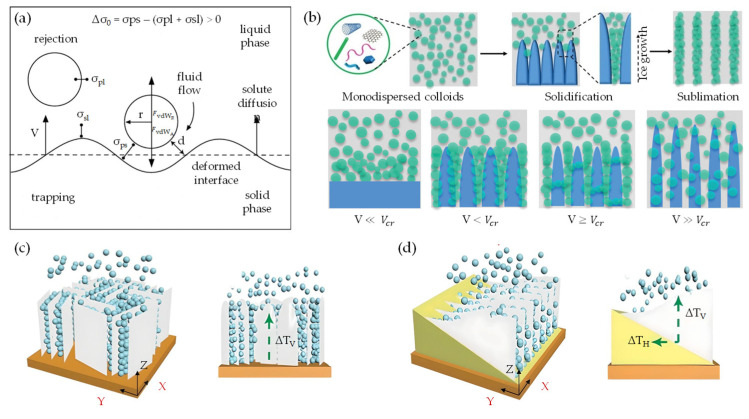
(**a**) Schematic illustration of the freezing front interactions with balancing vdW attractive and repulsive forces at the liquid–solid interface [217]. Reproduced with permission. Copyright: The Royal Society. (**b**) Representation diagram of freeze front progressing and ice growth with different velocities [19]. Modified with permission. Copyright: WILEY-VCH. (**c**,**d**) Schematic view illustrates freezing kinetics in terms of single–dual temperature gradient and vertical–horizontal directionality (△T_V_ and △T_H_) [219]. Reproduced with permission. Copyright: American Association for the Advancement of Science.

The process of freeze-casting [19] or ice-templating [216] techniques can be leveraged to fabricate quasi-3D nanoarchitecture. This is accomplished by solidifying a monodispersed colloid of quasi-2D graphene nanosheets and quasi-1D NC chain, followed by sublimating solvent under vacuum. The resulting structure is highly porous and can be stabilized through chemical or physical crosslinking functionalization. From a thermodynamic perspective, the liquid–solid phase transition is correlated with the interfacial free energy (∆σ_0_) (Figure 15a), as demonstrated within a single scale [19,217]. This indicates that ice-templating occurs when the solid phase is rejected as the solidification front progresses, in accordance with Equation (12):

Δσ_0_ = σ_ps_ − (σ_pl_ + σ_sl_) > 0 (12)

where σ_ps_, σ_pl_, and σ_sl_ are the surface energies between particle–solid–liquid phases–interfaces, respectively [19]. Consequently, the energy equilibrium within a system experience vdW attraction ($F_{{\mathrm{vdW}}_{A}}$) and ($F_{{\mathrm{vdW}}_{R}}$) repulsion forces governed by the following Equations (13) and (14):

(13) $$F_{{\mathrm{vdW}}_{A}}=6\pi \eta vr^{2}/d$$

(14) $$F_{{\mathrm{vdW}}_{R}}=2\pi r^{2}{\left.\left(\;\frac{a_{0}}{d}\right.\right)}^{n}$$

where η represents the liquid dynamic viscosity, v signifies the freezing velocity, and *r* is the radius of the solid particle radius. The thickness of the liquid layer and the distance between the particle and the ice front are denoted by *d* and *a*_0_, respectively. Meanwhile, *n* corresponds to the correction applied to the repulsive forces acting on the particle. Moreover, nucleation and ice growth play a crucial role in the alignment of vdW heterointerfaces until the equilibrium is reached at the solidification stage, as illustrated in Figure 15b. This concept is derived from the critical interface velocity rates (V_cr_) of the ice front, as given in Equation (15).

(15) $$Vcr=\frac{\;{∆}_{\sigma_{0}}}{3\eta r}{\left.\left(\;\frac{a_{0}}{d}\right.\right)}^{n}$$

Therefore, the vdW attractive ($F_{{\mathrm{vdW}}_{A}}$) and repulsive ($F_{{\mathrm{vdW}}_{R}}$) forces are the dominant factors manipulating the freezing-front interactions within monodispersed colloids [19,60,218].

**Figure 16:**
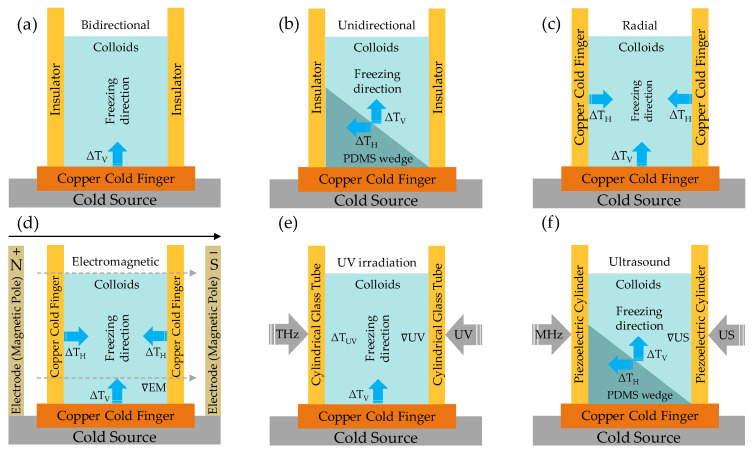
Proposed diagrams illustrating a multiscale design implementing ex situ alignment induced approaches for quasi-2D/1D monodispersed colloids during the solidification stage: (**a**) Bidirectional; (**b**) Unidirectional; (**c**) Radial; (**d**) Electromagnetic; (**e**) UV irradiation; and (**f**) Ultrasound.

Noticeably, as indicated by Equations (12)–(15), the multiscale of monodispersed colloids can be physically and chemically manipulated during the solidification stage, in conjunction with freezing kinetics (i.e., temperature gradient △T, directionality, and external force fields) [19,219]. However, as illustrated in Figure 15c,d, the colloidal suspension begin to freeze under single (△T_V_) or dual (△T_V_, △T_H_) temperature gradients, leading to ice nucleation originating randomly on the cold source surface [219]. As a result, single large-scale or multiple small-scale domains may be initiated (Figure 15c,d-right) per ice orientation and growth direction (X-Y-Z axis) in the vertical–horizontal plane [18,219]. Thus, specific cellular, lamellar, and honeycomb mesoporous nanoarchitecture could be achievable by controlling nucleation and ice growth at the early stages [16,220]. In addition, researchers have explored the applicability of bidirectional, unidirectional, and radial freeze casting to control nanoscale pore morphologies and geometric porous nanoarchitecture [13,17,18,19,23,30,219,221] upon sublimation and lyophilization stages. Built on this conception, it is worth noting that the removal of unnecessary surface functional groups at quasi-2D/1D heterointerfaces combined with ex situ alignment induced approaches may contribute to the formation of ordered symmetric, centrosymmetric, and asymmetric nanoarchitecture patterns [23]. These outcomes are derived from the chemical and physical crosslinking of monodispersed colloids that occur during the manipulation of freezing conditions [222], such as thermal gradients (ΔT_H_ and ΔT_V_) and directionality (Figure 16a–c) [219,223]. Furthermore, the implementation of external fields, such as electromagnetic [117,224], light irradiation [119], and ultrasound acoustic [118], can induce ex situ alignment directionality and orientation patterns of quasi-2D/1D at liquid–solid interfaces during solidification [19]. In the presence of an electromagnetic (EM) field (Figure 16d), adaptability stems from the inherent dipolar nature of quasi-2D/1D, along with the V_cr_ response at liquid–solid interfaces that varies with frequency-dependency by the directional flow of the ∇EM field intensity [225].

A critical aspect of these nano-entities is the highly anisotropic polarizability (polarizable π electrons and cation-π interaction) [226], which is determined by the geometric parameters (i.e., sp^2^ hybridized domains) and profoundly impacts behavior in the ∇EM field (parallel or perpendicular). Simultaneously, vdW forces arise due to temporary fluctuations in electron distribution (temporary dipole formation) [227,228], significantly contributing to the stability of the dual-aligned structure in close proximity. The dynamic interplay between these inherent driving forces and electrostatic ∇EM field induced forces [229] could dictate the nature of nano-entities alignment [230]. Alternatively, under exposure to an ultraviolet (UV) irradiation field, the occurrence of photothermal and photophoretic phenomena is observed due to the absorption and re-emission of ∇UV light by the quasi-2D/1D colloidal nano-entities [231,232,233]. This process prompts a temperature rise, thereby generating a thermal gradient (ΔT_UV_) within the proximate medium (Figure 16e). This ΔT_UV_ generation can potentially incite a thermophoretic displacement in quasi-2D/1D heterointerfaces, directing the orientation during the solidification stage. Additionally, the anisotropic nature of these vdW heterointerfaces [234] arises from fluctuation phenomena that manipulate the alignment under ΔT_UV_ thermo–photophoretic forces [235] along the UV light-propagation direction. This process drives nano-entities towards areas of lower ΔT_UV_ due to the natural diffuse tendency, referred to as photopheresis, which determines the alignment configurations. Likewise, acoustophoresis is a phenomenon that involves the alignment and manipulation of nano-entities using acoustic ultrasound (US) fields [118]. When quasi-2D/1D colloidal nano-entities are exposed to ∇US fields (Figure 16f), they experience acoustic radiation forces (F_US_ = −∇U_US_) that drive them towards regions of minimal ∇U_US_ potential energy [120]. The negative gradient indicates that the F_US_ is always directed from regions of higher potential energy (anti-nodes) to regions of lower potential energy (nodes). Through manipulation of external fields, such as ∇EM, ∇UV, and ∇US, researchers are able to precisely align sophisticated quasi-2D/1D nano-entities, thus enabling the fabrication of quasi-3D scaffolds with multidirectional architectural hierarchies [19,120,236,237]. These 3D macroscopic scaffolds exhibit a variety of porous structures, including cellular [18] or lamellar [16,238], honeycomb [17], and concentric ring-like [19,118].

## 6. Outlook

The roadmap from fundamental forces to the engineering of self-assembled architectures outlines an intriguing field in exploring assemblies driven by van der Waals (vdW) forces. These forces significantly contribute to forming anisotropic, quasi-2D graphene, and quasi-1D nanocellulose heterointerfaces, thus opening new avenues towards developing quasi-3D nanoarchitecture. The main point of this exploration is to understand the subtle but significant role of the synergistic interactions of vdW forces at the interface during the assembly process. The vdW distance and gap are central to this exploration, which plays a significant role in electrostatic stabilization. This necessitates precise control over these aspects to establish stable, well-defined nanostructures. However, due to the inherently weak nature of these forces, achieving this level of control poses a considerable challenge, underlining the need for pioneering research that offers precision and reliability. Moreover, the functionalization of these quasi-2D/1D heterointerfaces, both covalently and non-covalently, has emerged as a significant area of interest. By systematically manipulating anisotropic quasi-2D inter–intrasheet and quasi-1D inter–intrachain configurations, we could potentially manipulate the subsequent performance of the vdW-driven assemblies. Specifically, harnessing the dangling π-bonds for covalent interactions and engineering dangling bond-free structures for non-covalent interactions unveil new possibilities. Additionally, the prospect of in situ reduction mechanisms appears promising in simplifying the functionalization process. Still, the control and reproducibility challenges in these processes pose significant hurdles that future studies must address. Furthermore, the transition dynamics prompted by vdW forces, encompassing load, charge, and heat transfer, demand careful consideration due to their profound impact. Precisely, the interplay of in-plane and out-of-plane dynamics within these transitions brings another dimension of complexity, although our current knowledge is largely confined to mono–bi–trilayer systems. However, it is expected that a more in-depth understanding of these mechanisms across multilayer systems, whether obtained computationally or experimentally, could unlock prospects to leverage inherent properties needed by a broad range of applications. In line with this, adopting in situ and ex situ alignment induced strategies has become an increasingly burgeoning area of research interest in developing highly controlled and ordered nanoarchitecture. A comprehensive understanding of the dominant vdW forces and induced forces during directional solidification could make precise control over the single–dual-aligned configurations under neither internal nor external fields realizable. Looking forward, the prospects of vdW-driven assemblies in generation quasi-3D nanoarchitecture hinge on overcoming these challenges. These include exploring advanced functionalization techniques, comprehending the underlying dynamics of the transitions, and achieving controlled alignment synchronized with vdW forces. The potential applications of these assemblies are multifold, from advanced electronics to high-performance functional materials and beyond. In this respect, the roadmap from forces to assemblies is far from over; rather, we have only just begun to understand the profound implications of these vdW-driven assemblies and the exciting prospects they offer.

## Data Availability

Not applicable.
